# Extranodal Natural-Killer/T-Cell Lymphoma, Nasal Type

**DOI:** 10.1155/2010/627401

**Published:** 2010-12-30

**Authors:** Harinder Gill, Raymond H. S. Liang, Eric Tse

**Affiliations:** Division of Haematology, Medical Oncology and Bone Marrow Transplantation, Department of Medicine, University of Hong Kong, Queen Mary Hospital, Hong Kong

## Abstract

The World Health Organization (WHO) classification recognizes 2 main categories of natural killer (NK) cell-derived neoplasms, namely, extranodal NK/T-cell lymphoma, nasal type, and aggressive NK-cell leukaemia. Extranodal nasal NK/T-cell lymphoma is more frequent in the Far East and Latin America. Histopathological and immunophenotypical hallmarks include angiocentricity, angiodestruction, expression of cytoplasmic CD3 epsilon (**ε**), CD56, and cytotoxic molecules and evidence of Epstein-Barr virus (EBV) infection. Early stage disease, in particular for localized lesion in the nasal region, is treated with chemotherapy and involved-field radiotherapy. On the other hand, multiagent chemotherapy is the mainstay of treatment for advanced or disseminated disease. L-asparaginase-containing regimens have shown promise in treating this condition. The role of autologous hematopoietic stem cell transplantation is yet to be clearly defined. Allogeneic hematopoietic stem cell transplantation, with the putative graft-versus-lymphoma effect, offers a potentially curative option in patients with advanced disease.

## 1. Introduction

Natural killer (NK) cells are lymphoid cells that mediate lysis of tumor cells and bacteria- or virus-infected cells and the production of immunomodulatory cytokines [1]. Morphologically, mature NK cells are large granular lymphoid cells, which are characterized by the presence of pale cytoplasm containing azurophilic granules. Unlike T-cell large granular lymphocytes, they are negative for surface CD3 but characteristically express cytoplasmic CD3 epsilon (*ε*), CD56, and cytotoxic molecules. Furthermore, clonal rearrangement of the T cell receptor (TCR) genes is also absent in NK cells. The latest World Health Organization (WHO) classification recognizes 2 main categories of NK cell-derived neoplasms, namely, aggressive NK cell leukemia and extranodal NK/T-cell lymphoma, nasal type [2, 3]. Evidence of Epstein-Barr virus (EBV) infection in the lymphoma cells is a consistent finding which may be directly involved in lymphomagenesis [4–6]. NK/T-cell lymphoma of the nasal type has a distinctive ethnic and geographical distribution, accounting for 7% to 10% of all non-Hodgkin lymphomas in Asia and Latin America, but only 1% of that amongst Caucasians [7–9]. On the other hand, aggressive NK-cell leukaemia, which is considered as the leukaemic counterpart of NK/T-cell lymphoma, is an uncommon disease. The rest of this paper will focus the discussion on NK/T-cell lymphoma of the nasal type.

## 2. Histopathology

**Figure 1:**
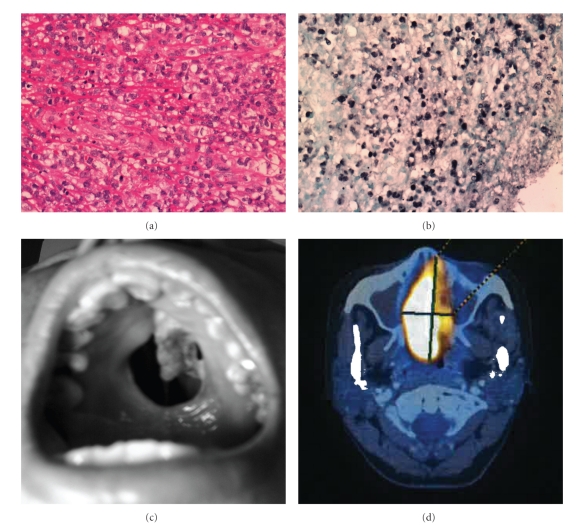
(a) Histology of NK/T-cell lymphoma showing presence of atypical lymphoid cells, which are medium to large in size with irregular and hyperchromatic nuclei. (b) *In situ* hybridization for Epstein Barr virus-encoded early RNAs (EBER) positivity in NK/T-cell lymphoma. (c) NK/T-cell lymphoma of the nasal type presenting with perforation of hard palate. (d) PET/CT scan of a patient with NK/T-cell lymphoma showing a FDG-avid mass in the right nasal cavity.

NK/T-cell lymphoma shows an angiocentric and angiodestructive pattern of growth with associated geographical necrosis and ulceration. Coagulative necrosis and apoptotic bodies are frequently encountered. The tumor cells are small to medium in size with occasional large and anaplastic forms (Figure 1(a)). The lymphoma cells may be admixed with a polymorphic infiltrate of nonneoplastic inflammatory cells including small lymphocytes, plasma cells, histiocytes, and eosinophils. The immunophenotype of NK lymphoma cells is classically positive for CD2, CD56, and cytoplasmic CD3 epsilon (*ε*). They are negative for surface CD3. Unlike normal NK cells, the tumor cells are usually negative for CD7 and CD16. They express cytotoxic granule associated proteins granzyme B, T-cell restricted intracellular antigen (TIA-1), and perforin. Presence of EBV infection shown by *in situ* hybridization (ISH) for EBV-encoded early small RNA (EBER) is a distinctive feature (Figure 1(b)). The World Health Organization classification requires both EBV positivity and expression of cytotoxic granules for the diagnosis of NK/T-cell lymphoma, nasal-type [2]. EBV positive NK/T lymphomas with typical clinical and morphological features and expression of cytotoxic granules can be classified as such even if they deviate from the classical immunophenotype, for instance, CD8 positivity or CD56 negativity [10]. The classification of EBV negative tumors with expression of cytotoxic proteins remains controversial. Occasional neoplastic cells can express CD30 with large cell morphology simulating anaplastic large cell lymphoma [10]. Aberrant expression of the B-cell marker CD20 has been described in 3 cases to date but the significance of which remains uncertain [10–12].

## 3. Clinical Features

NK/T-cell lymphomas have a distinctive geographical distribution with most cases being reported in Asia and Latin America. Clinically, it is useful to classify NK/T-cell malignancies into two categories, namely, nasal NK-cell lymphomas, and nonnasal or extranasal NK/T-cell lymphomas, depending on the site of the lesions [13]. Nasal NK/T-cell lymphomas occur in the nose and the upper aerodigestive tract. Males predominate and they usually present in their fifties. Common sites of involvement include the nasal cavity, nasopharynx, paranasal sinuses, hypopharynx, and larynx [10, 13]. As a result, the common presenting symptoms include nasal obstruction, epistaxis, and facial swelling. Retro-orbital involvement causes proptosis and impairment of extraocular movement. Occasionally local extension from the nasal cavity causes destruction of the hard palate with the characteristic midline perforation, previously referred to as “lethal midline granuloma” (Figure 1(c)). Bone marrow involvement occurs in less than 10% of patients and distant metastasis is unusual.

Extranasal or nonnasal NK/T-cell lymphomas likewise are more prevalent in males in their fifth decade. As its name implies, it occurs outside the typical nasal region, and the common primary sites involved include the skin, gastrointestinal tract, salivary glands, spleen, and testis. Unusual sites of involvement such as the muscle, the adrenal gland, and the female genital tract (ovaries and uterus) have been reported [10]. Distant dissemination occurs early in the clinical course of the disease. Many extranasal NK/T-cell lymphomas might represent disseminated nasal NK/T-cell lymphomas, particularly when the primary sites of extranasal NK-cell lymphomas are precisely the sites where nasal NK/T-cell lymphomas will metastasize to. Occult nasal involvement should therefore be carefully evaluated by flexible nasal panendoscopy with multiple random biopsies in patients presenting with extranasal NK/T-cell lymphoma. In both nasal and nonnasal NK/T-cell lymphomas, isolated nodal involvement is highly unusual [10, 13].

## 4. Staging

A standard staging system for NK/T-cell lymphomas is lacking. As for other extranodal lymphomas, the Ann-Arbor staging system, originally designed for Hodgkin's lymphoma, is unsatisfactory for NK/T-cell lymphomas [14]. It does not take into account the tumor size and the resultant invasion to contiguous structures which may be an important prognostic determinant. A T-staging system, originally designed for sinonasal B-cell lymphoma has been adopted to overcome this problem by taking into account the extent of local tumor involvement [15]. T1 denotes confinement to the nasal cavity. T2 indicates extension to the maxillary antra, anterior ethmoid sinus or hard palate. T3 indicates extension to posterior ethmoid sinus, sphenoidal sinus, orbit, superior alveolar bone, cheeks, or superior buccinators space. T4 indicates involvement of the inferior alveolar bone, inferior buccinators space, infratemporal fossa, nasopharynx, or cranial fossa. Patients with T1/2 disease had shown a better clinical outcome than those with T3/4 disease [16].

## 5. Imaging Assessment

Computerized tomography (CT) has been conventionally utilized to assess the local extent of the disease as well distant metastases. Magnetic resonance imaging (MRI) has been shown to better define local soft tissue and bony involvement. The use of Fluorine-18 fluorodeoxyglucose positron emission tomography computerized tomography (18-FDG PET-CT) has been evaluated (Figure 1(d)). NK-cell lymphomas were shown to be FDG-avid and PET-CT offers more accurate definition of the extent of involvement by distinguishing lymphoma involvement from inflammatory masses [17]. This has important implications in radiotherapy planning. PET, however, may not detect morphologically occult marrow infiltration uncovered by ISH for EBER [17]. In a recent retrospective analysis of 117 patients with non-Hodgkin lymphomas, the highest maximum standard uptake value (SUV_max_) of NK-cell lymphoma was 9.2 ± 4.5 which is significantly lower than that of aggressive B-cell lymphomas but high than that of indolent B-cell lymphomas [18]. 18-FDG PET-CT is increasingly used both at diagnosis and for monitoring of response to therapy.

## 6. Quantification of Plasma EBV DNA Level

Apoptosis of proliferating EBV-related tumor cells releases EBV DNA into the circulation [19]. Quantification of circulating plasma EBV DNA level by quantitative polymerase chain reaction (PCR) is used as a surrogate marker of tumor burden and has been shown to correlate with disease status in patients with NK/T-cell lymphomas. A high level of circulating plasma EBV DNA has correlated with high tumor load, extensive disease, poorer response to treatment, and inferior survival [20].

## 7. Prognostic Factors

The International Prognostic Index (IPI) predicts outcome in nasal NK/T-cell lymphoma. Patients with IPI of 1 or less were shown to have a better overall survival [13, 14]. In a recent retrospective analysis of 172 patients with extranodal NK/T-cell lymphoma, nasal type, and aggressive NK-cell leukemia, 4 prognostic factors were identified. These include nonnasal type, stage, performance status, and number of extranodal sites [21]. Level of circulating plasma EBV DNA at presentation has been shown to significantly affect disease-free survival [13, 14, 20]. Finally, a prognostic model based on a retrospective analysis of 262 patients has been proposed by Korean investigators [22]. In this Korean prognostic model, four prognostic groups have been identified depending on B-symptoms, stage, LDH level, and regional lymphadenopathy.

## 8. Treatment of Localized Disease

Because of the low incidence of NK/T-cell malignancies, randomized control trials on treatment have not been available. Most treatment protocols are instead consensus based and are derived from retrospective and small prospective studies. The prognosis of nasal NK/T-cell lymphomas has gradually improved with modern treatment protocols combining chemotherapy and radiotherapy. On the other hand, prognosis remains guarded in most extranasal and disseminated cases. 

For stage I/II nasal NK/T-cell lymphomas, radiotherapy is an important modality of treatment. Nonnasal NK cell lymphomas tend to have advanced or disseminated disease at presentation and the role of radiotherapy is often limited. The reported overall response rate of localized nasal disease after radiotherapy ranged from 60% to 80% with a complete remission (CR) rate of 40% 80% and a 5-year overall survival (OS) of 40 to 59% [13, 14]. Careful planning with the assistance of modern imaging modalities like CT and MRI is required before radiotherapy. A total radiotherapy dose of 30 to 60 Gy with fractional doses of 1.5 to 2.5 Gy is typically used [14]. Despite the excellent initial response to radiotherapy alone, a high relapse rate of around 50% is reported. Local failure, including in-field and margin failures, usually occur within 1 year and is associated with a radiotherapy dose of less than 45 to 50 Gy and radiotherapy planning not assisted by imaging [23]. Local relapses beyond 2 years are uncommon although late relapses have been reported [23, 24]. Systemic relapses occur in 25% to 30% of cases, more than half of which is not associated with local recurrences [13]. In light of the high relapse rate with radiotherapy alone, combination of chemotherapy and radiotherapy is the current standard of care in patients who can tolerate systemic treatment. A commonly used anthracycline-containing regime is CHOP (cyclophosphamide, doxorubicin, vincristine, and prednisolone). Primary chemotherapy for stage I/II nasal NK/T-cell lymphomas yields a CR rate of around 40% to 60%. Nevertheless, a high rate of disease progression (30–40%) and relapse after initial CR (30–40%) is observed [13, 14]. The expression of multidrug resistance (MDR) gene and high levels of P-glycoprotein in NK lymphoma cells underlies the resistance to anthracyclines and vinca alkaloids [25]. Regimens bases on non-P glycoprotein efflux chemotherapeutic agents such as L-asparaginase, ifosphamide, and methotrexate may potentially improve response rates. The optimal dose, combination and sequence of radiotherapy and chemotherapy remain to be defined though the current trend is to provide involved field radiotherapy after an initial three courses of chemotherapy. Concurrent chemoradiotherapy has been studied in two recent phase II trials with favorable results [26, 27]. In our center, for patients with stage I/II disease, the standard is to offer multiagent chemotherapy using the regimen SMILE (*d*examethasone, *m*ethotrexate with leucovorin, *i*fosfamide, *L*-asparaginase, and *e*toposide). A total of six courses of chemotherapy is offered. Involved-field radiation is administered after 3 courses of chemotherapy.

## 9. Treatment of Disseminated Disease

For the stage III/IV nasal NK/T-cell lymphomas, extranasal NK/T-cell lymphomas and aggressive NK-cell lymphoma/leukemia, chemotherapy is the primary treatment [13, 14]. Results from chemotherapy adopted from treating aggressive B-cell lymphomas such as CHOP is far from satisfactory [14]. L-asparaginase-containing regimens have shown promise. As mentioned early, L-asparaginase is not affected by P-glycoprotein. Furthermore, the tumor cells lack L-asparagine synthetase and are susceptible to L-asparaginase which depletes L-asparagines in NK lymphoma cells [14]. A retrospective analysis of Chinese patients with relapsed or refractory NK/T-cell lymphoma, nasal type yielded an overall response rate of 82% and a CR rate of 56%. The 5-year survival reported in that series was 67% [28]. The protocol SMILE (consisting Steroids, Methotrexate and folinic acid, Ifosfamide, L-asparaginase, and Etoposide) was studied in treating patients with relapsed and refractory disease. Prophylactic use of granulocyte colony-stimulating factor (G-CSF) was necessary due to significant marrow suppression. The overall response rate was 67% with a CR rate of 50% [29]. In our center, patients with advanced-stage disease are treated with 6 courses of SMILE chemotherapy regime. Allogeneic hematopoietic stem cell transplantation is also considered for suitable candidates (see below).

## 10. Role of Hematopoietic Stem Cell Transplantation (HSCT)

The unsatisfactory treatment outcome for advanced and disseminated NK/T-cell lymphoma has led to the use of high-dose chemotherapy and hematopoietic stem cell transplantation. Autologous HSCT has been evaluated in stage I/II disease in first or second complete remission, or chemosensitive relapse, and primary or secondary refractory disease without marrow involvement [13]. The disease status pre-HSCT significantly affected overall survival and patients with refractory disease had a significantly inferior survival after autologous HSCT [30]. As most patients with stage I/II disease would be expected to have durable remissions following multiagent chemotherapy and radiotherapy, the definite advantage of autologous HSCT in first complete remission (CR1) is questionable. However, based on a retrospective analysis on a small group of patients, there was evidence that autologous HSCT may be beneficial in a subgroup of patients in CR1 who have a high risk of relapse as determined by prognostic modeling [31]. HSCT is generally indicated in lymphoma patients achieving second complete remission (CR2) though further controlled trials are required to examine whether this also applies to NK/T-cell lymphoma. There is no survival advantage of autologous HSCT in patients with advanced or refractory disease [30]. Allogeneic HSCT, with the potential benefit of graft-versus-lymphoma (GVL) effect, is a sound option for patients with advanced disease. The GVL effect is further enhanced by the expression of EBV antigen on tumor cells, providing an alloreactive target [14]. Small series have shown that it is a potentially curative option [32]. In our centre, allogeneic haematopoietic stem cell transplantation is offered to selected cases with advanced stage disease who achieve complete remission after 6 courses of SMILE chemotherapy.
